# Prevalence of hearing loss and tinnitus in a group of adults with Human Immunodeficiency Virus

**DOI:** 10.4102/sajcd.v67i1.631

**Published:** 2020-02-04

**Authors:** Alison Millar, Karin Joubert, Alida Naude

**Affiliations:** 1 Department of Speech Pathology and Audiology, University of the Witwatersrand, Johannesburg, South Africa; 2 Ndlovu Wits Audiology Clinic and Outreach Programme, Dennilton, South Africa; 3 Centre for Augmentative and Alternative Communication, Faculty of Humanities, University of Pretoria, Pretoria, South Africa

**Keywords:** Human Immunodeficiency Virus, Acquired Immunodeficiency Syndrome, antiretroviral, hearing loss, tinnitus, audiology, rural

## Abstract

**Background:**

The Human Immunodeficiency Virus (HIV) has become a global pandemic. With the improvement of antiretroviral (ARV) treatment regimens, life-expectancy of HIV-positive individuals has increased. HIV literature suggests that head and neck manifestations may be the first indication of supressed immunity. Therefore, research regarding the effects of HIV and new treatment regimens on auditory function remains a priority.

**Objectives:**

To describe the audiological characteristics and determine the prevalence of hearing loss and tinnitus in a group of HIV-positive individuals on ARV treatment residing in a rural province.

**Methods:**

The study employed a cross-sectional descriptive research design. Participants were recruited from the clinic and pharmacy waiting areas of a medical centre in a rural area of Limpopo province, South Africa. Two participant groups, an HIV-positive group (*N*_1_ = 60) and an HIV-negative group (*N*_2_ = 32) were included in the study. The test battery comprised a comprehensive case history and a routine audiological test battery, which included otoscopy, tympanometry and pure tone audiometry (250 Hz to 8000 Hz).

**Results:**

No statistically significant difference was found regarding the prevalence of hearing loss in the two participant groups (*p* = 0.709). However, the prevalence of tinnitus was significantly higher in the HIV-positive group (*p* = 0.05).

**Conclusion:**

The insignificant difference in the audiological test battery results found between the two participant groups may be due to improved ARV treatment regimens and management strategies employed at the medical centre. However, the increased prevalence of tinnitus in the HIV-positive group may also be attributed to the ARV regimen and/or the result of subtle damage to the auditory system, which was not identified by the current audiological test battery. More insight may be obtained about the effects of HIV on hearing by employing a longitudinal research design and inclusion of a more ototoxicity sensitive test battery.

## Introduction

The Human Immunodeficiency Virus (HIV) is one of the most significant pandemics of modern times (Piot & Quinn, 2013). Although HIV is a global problem, 33.5% of people living with HIV can be found in sub-Saharan Africa, of which 19.4 million reside in the eastern and southern regions (UNAIDS, 2017). In 2002, HIV prevalence in South Africa was estimated to be 4.25 million, increasing to 7.52 million in 2018. However, when considering population growth, there has been an overall decline in incidence rate from 1.88% in 2002 to 1.21% in 2018 (Statistics South Africa, 2018).

The highest HIV burden in South Africa is reported in rural areas (Gaede & Versteeg, 2011). Limpopo, one of South Africa’s most rural provinces, has a reported prevalence of 17.2% (Human Sciences Research Council, 2017). High HIV prevalence in rural areas may be attributed to, amongst other factors, poor socioeconomic conditions, high unemployment rates and limited access to healthcare services and resources. These challenges adversely affect healthcare provision, which ultimately result in poorer healthcare outcomes for residents in these areas (Gaede & Versteeg, 2011).

HIV disease has, since the use of antiretrovirals (ARVs), changed from life-threatening to a manageable condition (Heinze, Swanepoel, & Hofmeyr, 2011). HIV targets the immune system by reducing cluster of differentiation 4 cells (CD4+) or T-lymphocytes (T-cells) (World Health Organization (WHO), 2015). An HIV-positive individual may transition through various stages of infection (categories 1 to 3 and A, B and C) based on the CD4+ count and presence of comorbid conditions. The most advanced stage of infection is known as Acquired Immunodeficiency Syndrome (AIDS) (Centres for Disease Control and Prevention (CDC), 1992). In the absence of ARV treatment, immune function remains supressed and HIV-positive individuals become more susceptible to the development of certain cancers and opportunistic infections (WHO, 2015).

Clinical manifestations involving the head and neck region have been reported as among the first indications of suppressed immunity (Lacovou, Vlastarakos, Papacharalampous, Kampessis, & Nikolopoulos, 2012). The reported prevalence of complications in the auditory system range from 14% to 75% (Chandrasekhar et al., 2000; Khoza & Ross, 2002; Prasad, Bhojwani, Shenoy, & Prasad, 2006; Van der Westhuizen, Swanepoel, Heinze, & Hofmeyr, 2013; Zuniga, 1999). Symptoms indicating involvement of the auditory system may include hearing loss, tinnitus, sensation of aural fullness, otalgia, otorrhoea, headaches, oscilliopsia, nausea and a variety of balance disturbances presenting as vertigo, dizziness, gait instability or disequilibrium (Khoza-Shangase & Van Rie, 2016; Zingler et al., 2007). These symptoms may be the result of direct damage to certain portions of the auditory and/or vestibular pathway or secondary damage resulting from opportunistic infections or use of ototoxic treatment regimens (Stearn & Swanepoel, 2010).

Various changes have been made to the HIV treatment guidelines since it was first published by the WHO in 2002. The most notable changes include earlier treatment initiation, the use of safer and less toxic ARV drugs and the inclusion of a single, fixed-dose combination tablet taken only once-daily (OD), which may improve treatment adherence (WHO, 2013, 2015). A variety of studies have suggested that ARVs may be ototoxic (Kakuda, 2000; Khoza-Shangase, 2011; Marra et al., 1997; Mata, Yebra, Tutor de Ureta, Villarreal, & García, 2000). However, establishing the ototoxic relationship between ARVs and auditory function is intricate due to a range of confounding variables affecting auditory function (Khoza-Shangase, 2011). These variables may include age (very young or aged >60 years); genetic susceptibility; duration of treatment; dosage; simultaneous use of diuretic therapy; co-treatment with other ototoxic drugs; previous history of receiving ototoxic medications or any sensitivity to these medications; impaired renal or hepatic function; pre-existing hearing loss; and history of noise exposure (Bauman, 2003). Contrary to the studies suggesting ototoxicity, a longitudinal prospective study conducted by Schouten and colleagues (2006) suggested that ARVs had no damaging effect on hearing. These authors further suggested that auditory function may be affected by HIV itself; however; hearing may be improved once immune function is strengthened with the use of ARV treatment. A therapeutic role of ARVs has also been suggested by Cohen and colleagues (2012), who reported that these agents had no negative effect on vestibular function. Direct comparison of the results from various ototoxic studies is however difficult, as different test batteries were employed, and thus different portions of the auditory system were evaluated.

As a result of improved ARV protocols and access to treatment, the life expectancy of HIV-positive individuals has increased (Swanepoel & Louw, 2010). Therefore, research regarding the effects of HIV and new treatment regimens on the auditory and vestibular system remains crucial.

### Objectives

The objectives of this study were to describe the audiological characteristics and determine the prevalence of hearing loss and tinnitus in HIV-positive individuals treated with ARVs residing in a rural community.

## Methods

A cross-sectional, descriptive research design was used to describe the audiological characteristics and determine the prevalence of hearing loss and tinnitus. Participants were recruited from the Ndlovu Medical Centre (NMC) situated in Elandsdoorn, a rural settlement in the Elias Motsoaledi Local Municipality of the Sekhukhune District (Limpopo province, South Africa). This non-governmental organisation has provided free integrated Primary Health Care to HIV-positive individuals since 1994 (Ndlovu Care Group, 2015).

### Participants

The first group comprised HIV-positive individuals (*N*_1_ = 60) and the control group, HIV-negative individuals (*N*_2_ = 32) (Table 1). Participants were aged between 18 and 50 years. The age limit was set at 50 years to eliminate the possible impact of the natural ageing process on the auditory system (Huang & Tang, 2010). All participants in the study were black Africans and the majority were Sepedi first language speakers. Most of participants resided in the Sekhukhune district and were from a disadvantaged socioeconomic background.

**TABLE 1:** Participants’ description.

Participant group	*N*	Age (in years)			Gender			
		Mean	Range	SD	Male		Female	
					*n*	%	*n*	%
HIV-positive	60	41.4	23–50	5.3	20	33	40	67
Control	32	32.5	18–50	9.1	9	28	23	72

SD, standard deviation.

The 1992 revised CDC classification system was used to determine the progressive stage of infection of participants in the HIV-positive group (Appendix 1). Most participants in the HIV-positive group (58%; *N*_1_ = 35) were in category A, with a further 18% (*N*_1_ = 11) in category B, and the remainder (23%; *N*_1_ = 14) in category C. Participants were on a wide variety of ARV protocols, with 88% (*N*_1_ = 53) on first-line ARV regimens. Of these, 50% (*N*_1_ = 30) were on a first-line fixed-dose combination treatment consisting of Efavirenz, Emtricitabine and Tenofovir disoproxil fumarate.

### Data collection protocol

Participants were recruited from the clinic and pharmacy waiting areas of the NMC using a purposive sampling strategy. Only participants who gave written consent were included in the study.

Data collection protocol comprised a review of HIV-positive participants’ medical records (CD4 count, presence of comorbid conditions, ARV protocol and treatment duration) to guide the classification of HIV-positive participants according to the CDC category. For the two participant groups, a comprehensive case history (medical and audiological) and routine audiological examination were conducted. The audiological test battery was conducted on site at the Ndlovu Wits Audiology Clinic. All equipment was calibrated prior to the commencement of the study (SABS 0154-1; 0154-2). The test battery included otoscopy (Heine Mini 3000 otoscope), tympanometry using a 226 Hz frequency probe tone (Interacoustics Titan tympanometer) and standard pure tone audiometry (Interacoustics Diagnostic Audiometer: AD629). A normal type A tympanogram was classified according to the following criteria: ear canal volume: 0.8 to 2.0 mL; static compliance: 0.3 to 1.8 mL; middle ear pressure: -100 daPa to +50 daPa (Jerger, 1970). Air conduction thresholds were obtained via headphones (250 Hz to 8000 Hz) for both ears. The air conduction thresholds obtained at 500 Hz, 1 kHz and 2 kHz were used to calculate the Pure Tone Average (PTA). Bone conduction testing was conducted when air conduction thresholds exceeded 25 dB HL at any frequency between and including 250 Hz and 4000 Hz. These thresholds were obtained by placing the bone vibrator on one mastoid at a time, in the case of a bilateral impairment, or one side only in the case of a unilateral impairment. Pure tone audiometry results were classified as within normal limits based on a PTA of ≤ 25 dB HL. Therefore, a PTA of > 25 dB HL was considered a hearing loss. The hearing loss was categorised as conductive, sensorineural, mixed or a hearing loss at only a specific frequency (e.g. abnormal threshold **of** > 25 dB HL at 8 kHz).

### Data analysis

The data analysis was performed using SAS Release 9.4 (SAS Institute Inc., Carey, NC, USA). Descriptive statistics were used to describe measures of central tendencies (mean value) as well as measures of variability (i.e. range and standard deviation [SD]). The Fisher’s exact test was used to establish significance of percentages between the two participant groups. This test was also used to establish whether prevalence of tinnitus between the various CDC categories (1–3) was significant. For probability tests, *p* ≤ 0.05 was considered statistically significant.

### Ethical consideration

Data collection commenced once ethical clearance was obtained from the Human Research Ethics Committee (Medical) of the University of the Witwatersrand (Protocol number: M160150) and permission was granted by the Limpopo Department of Health and Social Development.

## Results

### Audiological characteristics

Self-reported auditory symptoms (such as hearing loss, otalgia and a blocked-ear sensation) were reported in 33% (*N*_1_ = 20) of HIV-positive participants and 25% (*N*_2_ *=* 8) of the control group. Difference in the prevalence of these symptoms between the two groups was not statistically significant (*p* = 0.481; Fisher’s exact test).

**FIGURE 1:**
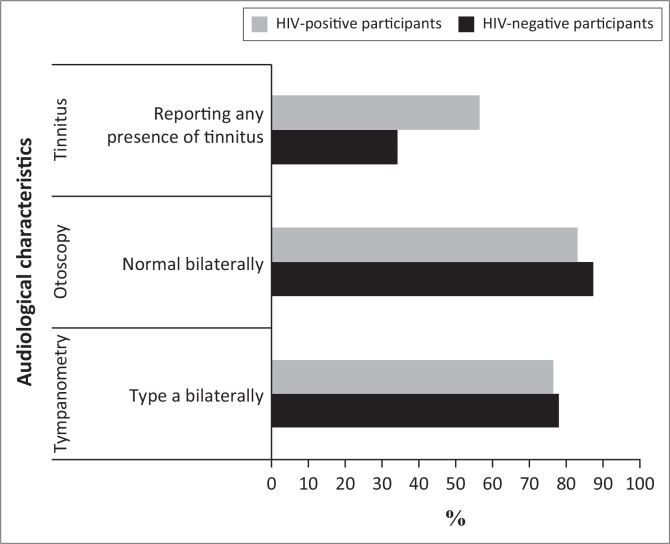
Most prevalent audiological characteristics in the two participant groups (HIV-positive participants [*N*_1_ = 60] and HIV-negative participants [*N*_2_ = 32]).

A majority of participants in both groups presented with normal otoscopy, tympanometry (Figure 1) and pure tone results with hearing < 30 dB HL bilaterally. Pure tone results were symmetrical in 83% (*N*_1_ = 50) of HIV-positive participants and 91% (*N*_2_ = 29) of the control group. A summary of the presence and nature of hearing loss in participants of the current study are presented in Table 2.

**TABLE 2:** Presence and nature of hearing loss.

Type of hearing loss	HIV-positive group (*N*_1_ = 60; 120 ears)				HIV-negative group (*N*_2_ = 32; 64 ears)			
	Left ear		Right ear		Left ear		Right ear	
	%	*n*	%	*n*	%	*n*	%	*n*
Conductive hearing loss	0	0	2	1	0	0	3	1
SNHL	2	1	2	1	6	2	13	4
Mixed hearing loss	7	4	3	2	0	0	0	0
Hearing loss (>25 dB HL) only at specific frequencies	8	5	8	5	3	1	3	1

SNHL, Sensorineural hearing loss.

The prevalence of abnormal otoscopy between the two groups was not statistically significant (*p* = 0.177; Fisher’s exact test), and neither was abnormal middle ear function, as suggested by tympanometry (*p* = 1.000; Fisher’s exact test).

### Prevalence of hearing loss

The mean PTA (dB HL) was similar in both groups. The HIV-positive group obtained a mean PTA of 12.6 dB HL and 12.3 dB HL in the left and right ears respectively. In the control group, the PTA was 11.4 dB HL in the left ear and 13.7 dB HL in the right. The prevalence of hearing loss was 10% (*N*_1_ = 6) in the HIV-positive group and 6% (*N*_2_ = 2) in the HIV-negative control group. This difference was not significant (*p* = 0.709; Fisher’s exact test).

### Prevalence of tinnitus

Tinnitus was reported by participants of both groups (Figure 1). Bilateral high frequency, intermittent tinnitus was most frequently reported in both groups. Bilateral tinnitus was reported by 48% of HIV-positive participants (*N*_1_ = 29) and unilateral tinnitus was reported by only 8% (*N*_1_ = 5). The overall prevalence of tinnitus in the HIV-positive group was 57% (*N*_1_ = 34) and in the control group, 34% (*N*_2_ = 11). Tinnitus reported in the control group was bilateral in nature. This difference was statistically significant (*p =* 0.05; Fisher’s exact test). The prevalence of tinnitus between different CDC categories increased at more progressive stages of infection (CDC category 1 (50%); CDC category 2 (65%) and CDC category 3 (53.9%). However, based on the statistical analysis, this increase was not significant (*p* = 0.633; Fisher’s exact test).

## Discussion

### Prevalence of hearing loss

The 10% hearing loss (>25 dB HL) in HIV-positive individuals and 6% in the control group is lower than the 19.88% hearing loss prevalence in the general population as reported for the Elias Motsoaledi Municipality (Joubert & Botha, 2019). The findings of the current study do not suggest a high prevalence of hearing loss in HIV-positive individuals (Table 3) and are similar to the findings reported by Van der Westhuizen et al. (2013). The difference in findings may be attributed to the CDC category of participants, the test battery employed as well as the criteria used to classify hearing loss.

**TABLE 3:** Prevalence of hearing loss and tinnitus in the current study in comparison to previous studies.

Symptom	Current study (*n* = 60)			Chandrasekhar et al. (2000) (*n* = 51)			Khoza and Ross (2002) (*n* = 150)			Van der Westhuizen et al. (2013) (*n* = 200)		
	%	*n*	*p*	%	*n*	*p*	%	*n*	*p*	%	*n*	*p*
Hearing loss	10	6	-	29	15	0.0135*	23	35	0.0333*	14	28	0.5163
Tinnitus	57	34	-	26	13	0.0018*	23	35	<0.0001*	26	52	<0.0001*

* , Statistically significant (≤ 0.05 *p*-values were considered significant; Fisher’s exact test) when compared to the prevalence of the current study.

Most of the participants in the current study (58%; *N*_1_ = 53) were classified as CDC category A, suggesting that they were asymptomatic and did not present with any secondary conditions. Contrary to this, in the study conducted by Chandrasekhar and colleagues (2000), the majority of participants were in CDC categories B (38%) and C (44%), whilst the minority were classified as CDC category A participants (18%). Similarly, Khoza and Ross (2002) also reported that the majority (91%) of HIV-positive participants in their study presented with secondary conditions. The prevalence of significantly higher hearing loss reported by these studies (Table 3) may be attributed to the higher prevalence of secondary conditions and medications used to treat these conditions.

The comparable hearing thresholds between the two participant groups in the current study may pertain to the ARV regimen used. The majority of participants were on a first-line ARV regimen, which has been associated with less toxicity (WHO, 2013) and improved treatment adherence due to its once-daily dosing (WHO, 2016). Furthermore, all participants were treated according to new ARV guidelines where treatment is initiated much earlier, irrespective of CD4+ count or progressive stage of infection. In addition, the NMC, where the study was conducted, employs strict monitoring protocols involving regular blood tests as well as counselling sessions. Therefore, as more emphasis is placed on the follow-up care, treatment adherence levels may have been more optimal, ultimately contributing to reduced auditory damage. The prevalence of slightly higher hearing loss reported by an earlier study (14%; *n* = 28; Van der Westhuizen et al., 2013) may have been attributed to the fact that only participants in CDC category C were receiving ARVs. Thus, the therapeutic effect of ARVs may have limited the destructive effects of HIV on the auditory system in the current study. These findings have also previously been suggested (Cohen et al., 2012; Schouten et al., 2006).

The audiological test batteries used in previous studies included additional measures such as auditory brainstem response audiometry (Khoza & Ross, 2002) or otoacoustic emissions (OAEs) (Van der Westhuizen et al., 2013). These measures may have been more sensitive at detecting auditory damage. The prevalence of reduced hearing loss in the current study may also be related to the criteria used to classify hearing loss. For example, a previous study (Khoza & Ross, 2002) classified hearing loss as any frequency with a threshold *of* >25 dB HL, whilst the current study based the criteria on the PTA. However, if frequency-specific criteria were used in the current study, the prevalence of hearing loss would be higher (22%; *N*_1_ = 13). Furthermore, the reduced prevalence of SNHL in the current study may be explained by the fact that hearing loss which only affected specific higher frequencies (e.g. 8 kHz) was not classified as sensorineural in nature. This type of configuration was classified in a separate category, whilst other studies may have described such configuration as SNHL.

### Prevalence of tinnitus

The most significant finding of the current study was the increased prevalence of tinnitus in the HIV-positive group. This prevalence was also significantly higher than reported in previous studies (Table 3). The high prevalence in the current study may be attributed to the inclusion of more detailed tinnitus categories. Therefore, participants may have been more likely to report tinnitus even if it was only experienced intermittently or in a different pitch than the more common ‘high frequency ringing’. It may however be possible that the increased prevalence of tinnitus may be a result of subtle damage to the auditory pathway, which was not detectable with conventional audiometry.

Similar to the findings reported by Van der Westhuizen et al. (2013), the prevalence of tinnitus was higher in CDC categories 2 and 3 compared to CDC category 1. Although this increase was not statistically significant, this finding may represent progressive damage caused by HIV during more advanced stages of infection (Van der Westhuizen et al., 2013). As tinnitus may present as a symptom of hearing loss, it would be expected that self-reported hearing loss would also be more prevalent in the HIV-positive group. The finding that hearing loss was not reported as frequently as tinnitus, may be attributed to tinnitus being related to non-auditory pathologies, or if the tinnitus is related to hearing loss, the nature or configuration may be more discreet and not always recognised or reported by the individual (Khoza-Shangase & Van Rie, 2016). Although high-frequency tinnitus was reported by 50% (*N*_1_ = 4) of HIV-positive participants with high-frequency hearing loss, this type of tinnitus was also reported by HIV-positive participants with hearing that was within normal limits. As the pitch of tinnitus may represent the hearing frequency most affected (Castillo & Roland, 2007), the inclusion of high-frequency audiometry (>8 kHz) may have identified more participants presenting with hearing loss.

## Conclusion

Comparable audiological results were found in the two participant groups. However, the prevalence of tinnitus was more significant in the HIV-positive group. Results from the current study warrant the need for longitudinal research to obtain insight regarding the causal relationship between HIV, its treatment regimen and audiological characteristics. These studies, especially if conducted in rural settings, may consider the use of mHealth technologies such as smartphone-automated pure-tone audiometry. These tests may prove to be more time-efficient when used for regular audiological monitoring purposes, ultimately allowing more individuals to be tested in a shorter amount of time (Brittz, Heinze, Mahomed-Asmail, Swanepoel, & Stoltz, 2018). To increase the generalisability of results, future studies should include participant groups with similar sample sizes, which are age- and gender-matched. The inclusion of an ototoxicity sensitive test battery (e.g. otoacoustic emissions test or high-frequency audiometry of >8 kHz) may also be valuable to evaluate the effects of new ARV regimens, treatment adherence levels and effective follow-up care on hearing status. As tinnitus was a prevalent finding in the HIV-positive group, research should place more emphasis on this area.

## Appendix 1

**TABLE 1-A1:** CDC classification system for HIV-infected adults and adolescents.

Clinical category	Conditions
A	Asymptomatic: Acute HIV or persistent generalized lymphadenopathy (PGL)
B	Conditions that are attributed to HIV infection or indicate a defect in cell-mediated immunity Conditions that are considered to have a clinical course or management that is complicated by HIV infection. Examples include, but are not limited to, the following: ■ Bacillary angiomatosis ■ Oropharyngeal candidiasis (thrush) ■ Vulvovaginal candidiasis, persistent or resistant ■ Pelvic inflammatory disease (PID) ■ Cervical dysplasia (moderate or severe)/cervical carcinoma in situ ■ Hairy leukoplakia, oral ■ Herpes zoster (shingles), involving two or more episodes or at least one dermatome ■ Idiopathic thrombocytopenic purpura ■ Constitutional symptoms, such as fever (>38.5°C) or diarrhea lasting >1 month ■ Peripheral neuropathy
C	Bacterial pneumonia, recurrent (two or more episodes in 12 months) Candidiasis of the bronchi, trachea, or lungs Candidiasis, esophageal Cervical carcinoma, invasive, confirmed by biopsy Coccidioidomycosis, disseminated or extrapulmonary Cryptococcosis, extrapulmonary Cryptosporidiosis, chronic intestinal (>1 month in duration) Cytomegalovirus disease (other than liver, spleen, or nodes) Encephalopathy, HIV-related Herpes simplex: chronic ulcers (>1 month in duration), or bronchitis, pneumonitis, or esophagitis Histoplasmosis, disseminated or extrapulmonary Isosporiasis, chronic intestinal (>1-month in duration) Kaposi sarcoma Lymphoma, Burkitt, immunoblastic, or primary central nervous system Mycobacterium avium complex (MAC) or Mycobacterium kansasii, disseminated or extrapulmonary Mycobacterium tuberculosis, pulmonary or extrapulmonary Mycobacterium, other species or unidentified species, disseminated or extrapulmonary Pneumocystis jiroveci (formerly carinii) pneumonia (PCP) Progressive multifocal leukoencephalopathy (PML) Salmonella septicemia, recurrent (nontyphoid) Toxoplasmosis of brain Wasting syndrome caused by HIV (involuntary weight loss > 10% of baseline body weight) associated with either chronic diarrhoea (two or more loose stools per day for ≥ 1 month) or chronic weakness and documented fever for ≥ 1 month

*Source*: Adapted from Centres for Disease Control and Prevention (CDC). (1992). *Revised classification system for HIV infection and expanded surveillance case definition for AIDS Among Adolescents and Adults*. Retrieved from https://www.who.int/hiv/strategic/en/cdc_1993_hivaids_def.pdf

**TABLE 2-A1:** CDC classification system for HIV-infected adults and adolescents.

CD4* cell count	Clinical categories		
	A: Asymptomatic Acute HIV or PGL	B: Symptomatic Conditions, not A or C	C: AIDS-Indicator Conditions
(1) 500 cells/µL	A1	B1	C1
(2) 200-499 cells/µL	A2	B2	C2
(2) 200 cells/µL	A3	B3	C3

*Source*: Adapted from Centres for Disease Control and Prevention (CDC). (1992). *Revised classification system for HIV infection and expanded surveillance case definition for AIDS Among Adolescents and Adults*. Retrieved from https://www.who.int/hiv/strategic/en/cdc_1993_hivaids_def.pdf

CD 4*, cluster of differentiation 4+; μL, microliter.
